# The Impact of Zirconium Oxide Nanoparticles on the Mechanical and Physical Properties of Glass Ionomer Dental Materials

**DOI:** 10.3390/ijms26115382

**Published:** 2025-06-04

**Authors:** Faiza Amin, Syed Faraz Moin, Naresh Kumar, Muhammad Asif Asghar, Syed Junaid Mahmood, Paulo J. Palma

**Affiliations:** 1 Department of Science of Dental Materials, Dow Dental College, Dow University of Health Sciences, Karachi 74200, Pakistan; kumar.naresh@duhs.edu.pk; 2 Dr Zafar H Zaidi Center for Proteomics, University of Karachi, Karachi 75270, Pakistan; faraz.moin@uok.edu.pk; 3 Food and Feed Safety Laboratory/FMRRC, PCSIR Laboratories Complex, Karachi 75270, Pakistan; masifpcsir345@gmail.com; 4 Plastic & Polymer Section, Applied Chemistry Research Centre (ACRC), PCSIR Laboratories Complex, Karachi 75270, Pakistan; junaid070880@gmail.com; 5 Faculty of Medicine, Center for Innovation and Research in Oral Sciences (CIROS), University of Coimbra, 3000-075 Coimbra, Portugal; 6 Faculty of Medicine, Institute of Endodontics, University of Coimbra, 3000-075 Coimbra, Portugal

**Keywords:** glass ionomer cements, zirconium oxide nanoparticles, flexural strength, flexural modulus, Vickers microhardness, water sorption and solubility

## Abstract

Glass ionomer cements (GICs) have been clinically attractive dental restorative materials for many years and are widely used as luting, lining, and restorative materials. However, these materials still have limitations in terms of weak physio-mechanical properties. The aim of the study was to evaluate the effect of zirconium oxide nanoparticles (nano-ZrO_2_ particles) on the physical and mechanical properties of two commercially available GICs. Four groups were prepared for each material: the control group (without nanoparticles) and three groups modified by the incorporation of nanoparticles at 2, 5, and 7 weight% (wt%). Firstly, the morphology and size of the nanoparticles were evaluated via scanning electron microscopy (SEM) and X-ray diffraction (XRD). Secondly, flexural strength, flexural modulus, Vickers hardness, water sorption, and solubility were evaluated. The main effect plots revealed that the addition of nano-ZrO_2_ particles enhances flexural strength, flexural modulus, and water sorption of GICs at a 7 wt% concentration and Vickers hardness at a 2 wt% concentration. The SEM analysis clearly shows that the cracks became narrower with the addition of nano-ZrO_2_ particles, whereas these cracks were completely closed at 7% nano-ZrO_2_ particles. The findings of the study appear promising, and it is anticipated that the optimization of nano-ZrO_2_ particles may aid the development of improved materials for load-bearing restorations.

## 1. Introduction

Dental caries is the most prevalent chronic disease worldwide. Pain, apical periodontitis, abscess, and infection are some serious consequences of untreated dental caries. The commonly used restorative materials for caries management include dental amalgam, resin composite, and glass ionomer cements (GICs). All these materials have their own strengths and weaknesses [1]. In 1972, Wilson and Kent introduced GIC materials, which are also known as polyalkenoates [2]. They are considered smart biomaterials due to certain distinctive properties like biocompatibility, chemical bonding to teeth, dimensional stability, excellent marginal integrity, fluoride release and rechargeability, low coefficient of thermal expansion, resistance to microleakage, and less setting shrinkage [3,4,5]. However, brittleness and poor mechanical properties are the major drawbacks of the GICs. Due to their inferior mechanical properties, they cannot be used in high-stress-bearing areas. To improve the performance of these materials, several attempts have been made by researchers to utilize their beneficial effects. In 1983, Simmons made the earliest attempt by adding silver powder into a GIC powder component [6]. Cho et al. [7] incorporated resins into GICs and compared them with conventional GIC materials. They observed a low sensitivity toward moisture. The fracture toughness and microhardness of GICs were increased after the addition of hydroxyapatite by Yap et al. [8] and Lucas et al. [9]. Nevertheless, due to the inherent and unresolved conflict between strength and toughness in glass ionomer cements, many of these approaches failed to perform adequately in the oral cavity [10].

For many decades, in the field of health science, nanotechnology has become one of the most active research areas, and many revolutionary developments have been made [11]. The importance of these materials gained interest by researchers when they realized that the size of the particles was one of the important factors that dictate the properties of the materials [11]. Recently, many experimental studies have been carried out after incorporating a variety of nanoparticles (NPs) in GICs, such as titanium dioxide nanotubes [12] and their NPs, [13,14] aluminum [15], and nano-ZrO_2_ particles [16]. To increase strength, Gjorgievska et al. [17] incorporated TiO_2_, Al_2_O_3_, and ZrO_2_ NPs into conventional GICs [18]. Surface porosities were reduced by adding these NPs when evaluated via scanning electron microscopy [18].

Zirconia is a natural white color with three crystal forms: (1) a monoclinic phase (m-ZrO_2_) stable at room temperature, (2) tetragonal (t-ZrO_2_) phase (1100–2370 °C), and (3) cubic (c-ZrO_2_) phase (above 2370 °C) [19]. Moreover, zirconia exhibits antibacterial, antifungal, antioxidant, and anticancer properties [4,20]. Due to these properties, it has been widely used in dental applications like dental implants and the construction of crowns, bridges, and inserts [4]. Alobiedy et al. [4] reinforced conventional GICs with nano-ZrO_2_ particles and found that the mechanical strength was increased, except for the wear rate. In most of the literature, researchers utilize commercially available nano-ZrO_2_ particles and incorporate them into restorative materials. To the best of our knowledge, no study has yet reported the synthesis of ZrO_2_ NPs in the laboratory and investigated the effect of nano-ZrO_2_ at three different concentrations. Therefore, the present study aimed to synthesize and characterize the ZrO_2_ NPs and incorporate them into conventional GICs to evaluate their physio-mechanical properties, such as flexural strength, flexural modulus, microhardness, and water sorption and solubility with and without nano-ZrO_2_ particles at three different concentrations after 24 h of immersion in distilled water.

## 2. Results

### 2.1. Characterization of ZrO_2_ Nanoparticles

#### X-Ray Diffraction (XRD)

**Figure 1:**
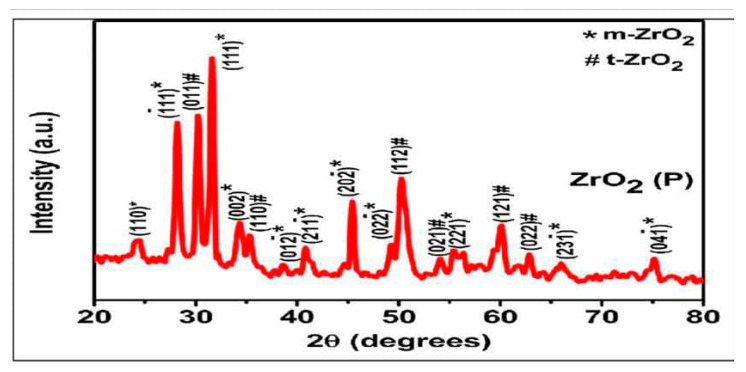
The graph demonstrates XRD pattern of nano-ZrO_2_ particles with monoclinic and tetragonal phases.

For particle size, the following equation was used, which is known as the Debye–Scherrer Formula.

(1) $$D=\frac{K\lambda }{\beta cos\theta }$$

where k is a constant and is assumed to be 0.9 for spherical particles; λ is wavelength of X-ray used—in this case, the value is 0.15406 nm; β is the FWHM (the full width of the selected peak at half maxima); and θ is the angle of the peak (Figure 1). The total number of peaks observed = 08, whereas the average particle size was found to be 13.7 nm. The XRD displayed a very strong peak at ~32° that corresponded to the 011 plane of ZrO_2_. Next, a peak at ~34° was due to the 002 plane of ZrO_2_, showing the monoclinic form of zirconia. Another smaller peak at ~49 corresponds to ZrO_2_ (020). Peaks through ~59–60° are those of 121 planes of ZrO_2_, indicating the tetragonal form of zirconium oxide nanoparticles. These findings are similar to the characterization studies published previously [21,22,23]. 

**Figure 2:**
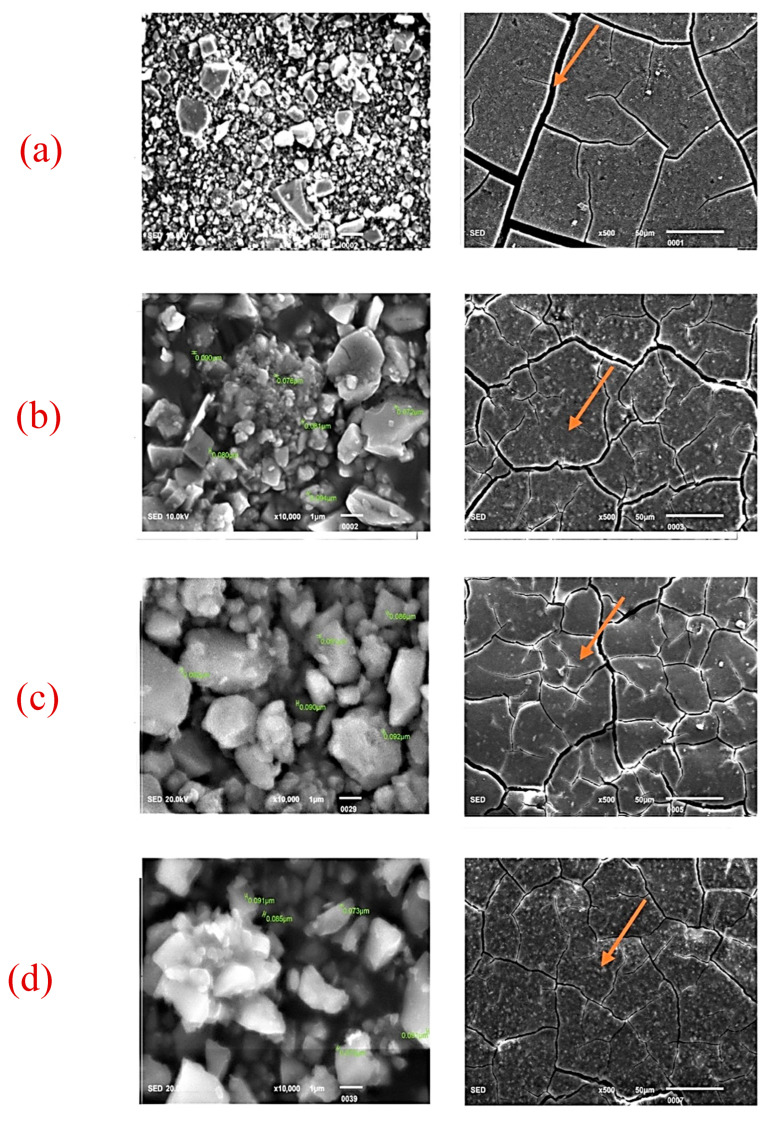
SEM images for GC GOLD LABEL 2 GIC (powder and disk). (**a**) Control (0%), (**b**) 2% nano-ZrO_2_ particles, (**c**) 5% nano-ZrO_2_ particles, and (**d**) 7% nano-ZrO_2_ particles. The disc form clearly depicts that the cracks were wider in control group (orange arrows), which then became narrower with the addition of nano-ZrO_2_ particles, whereas these cracks were completely closed on 7% nano-ZrO_2_ particles.

**Figure 3:**
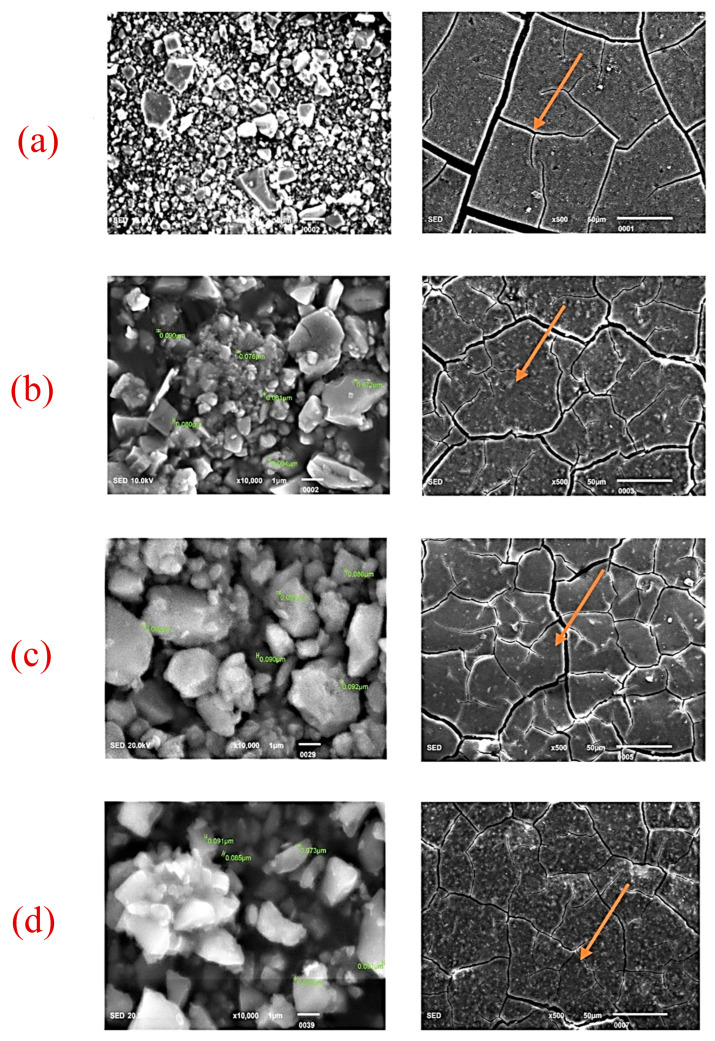
SEM images for SHOFU GIC (powder and disk). (**a**) Control (0%), (**b**) 2% nano-ZrO_2_ particles, (**c**) 5% nano-ZrO_2_ particles, and (**d**) 7% nano-ZrO_2_ particles. The disc form clearly depicts that the control group shows cracks, which were wider but were not improved by the addition of nano-ZrO_2_ particles (Figure 3a–d) (orange arrow).

**Figure 4:**
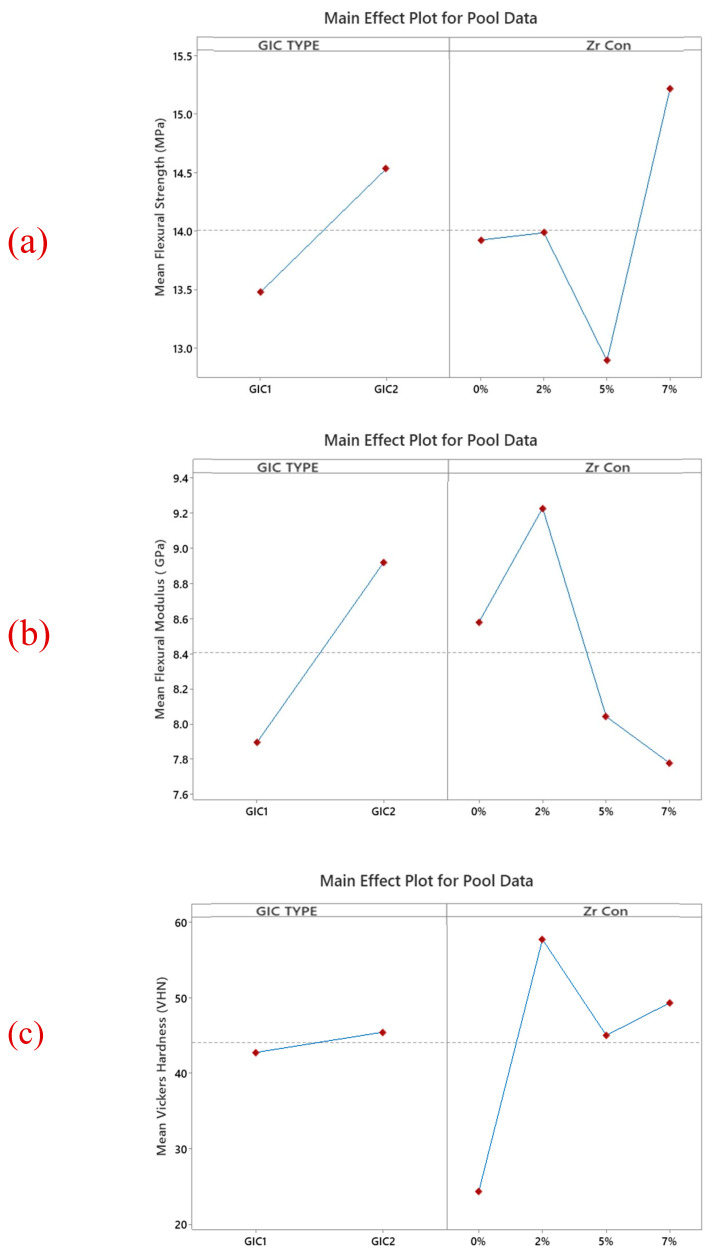
The main effects of the combined (**a**) flexural strength, (**b**) flexural modulus, and (**c**) surface hardness data, highlighting the effect of material type and nano-ZrO_2_ concentration. In general, GIC 2 appears to be stronger than GIC 1. Moreover, the addition of 7% nano ZrO_2_ shows an increase in the flexural strength and modulus and a decrease in the surface hardness of GICs.

**Figure 5:**
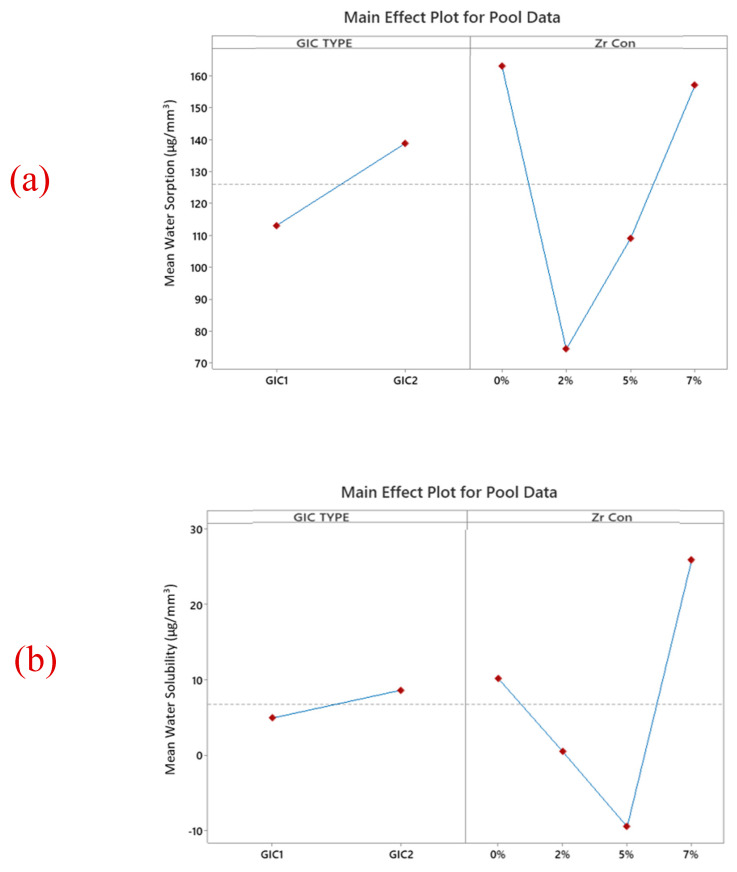
The main effects graphs of combined (**a**) water sorption (**b**) water solubility data highlight the effect of material type and nano-ZrO_2_ concentration. In general, GIC 1 has lower water sorption and solubility than GIC 2. Moreover, the addition of 7% nano ZrO_2_ shows decreased water sorption, whereas water solubility was increased.

The SEM images of ZrO_2_ and after the addition in GIC-1 and GIC-2 powder at different concentrations are given in Figure 2a–d and Figure 3a–d. The two-way ANOVA revealed that the flexural strength was affected by the material type (*p* = 0.018), and ZrO_2_ concentration (*p* = 0.007). The flexural modulus data were not affected by the material type (*p* = 0.182) and ZrO_2_ concentration (*p* = 0.371). The material type did not cause any significant influence on the surface hardness data (*p* = 0.419). However, the same characteristic was substantially influenced by the concentration of ZrO_2_ (*p* = 0.000). The water sorption showed a statistically significant difference regarding the ZrO_2_ concentration (*p* = 0.004), whereas it was not affected by the material type (*p* = 0.027). However, water solubility did not significantly affect both the material type (*p* = 0.190) and ZrO_2_ concentration (*p* = 0.948). Table 1 represents a positive impact of nano-ZrO_2_ particles on the mean flexural strength and mean Vickers microhardness GIC 1, whereas the mean flexural modulus was not affected by the incorporation of nano-ZrO_2_ particles. The means of water sorption and solubility after the incorporation of nano-ZrO_2_ particles in GICs are given in Table 2. Water sorption was found to be reduced at a 7% concentration in GIC 1, whereas no impact was observed at any concentration for water solubility. The effect of the material type and nano-ZrO_2_ particles on flexural strength, flexural modulus, and surface hardness data as a main effect graph is shown in Figure 4, whereas the main effects graphs of the water sorption and solubility are depicted in Figure 5.

## 3. Discussion

### 3.1. Flexural Strength

The ISO 9917-2 [24] protocol was followed to measure the flexural strength in this study [25]. We found that the incorporation of nano-ZrO_2_ particles enhanced the flexural strength of GIC 1 used in the current study. These smaller NPs act as additional bonding sites between the larger particles by occupying the empty spaces to reinforce the cement [26]. The crystalline nature of monoclinic and tetragonal nano-ZrO_2_ particles depicted by XRD around the amorphous matrix of the GIC might be the reason for the increase in strength [26]. The formation of a poly salt bridge by nano-ZrO_2_ particles between the GIC matrix improves the flexural strength of GIC 2 [27]. The results obtained from the current study were comparable to the results presented by Kutuz et al. [27] and Sajjad et al. [28]. They concluded that the effect of nano-ZrO_2_ particles was concentration-dependent, as the highest strength was found at 7%, followed by 5% and then 2% nano-ZrO_2_ particles in comparison with the control group. This was explained by preventing the crack propagation of the GIC when a local compression stress was developed due to a change in volume after tetragonal zirconia was transformed to monoclinic zirconia, increasing the fracture resistance of the material [29]. Nevertheless, this phase transformation in XRD was found to be consistent with as-received nanoparticles without undergoing any changes. In addition, when the authors calculated the particle size of the crystal by using XRD, it was found to be 13.7 nm, falling within the correct definition of nano-ZrO_2_ particles. Moreover, the control group of GIC 2 showed greater flexural strength when compared with GIC 1. The addition of nano-ZrO_2_ particles did not increase the flexural strength of GIC 2. Similar results were found when TiO_2_ NPs (3 and 5%) were added to conventional GICs by Garcia-Contreras et al. in 2015 [30]. They explained that this finding might be due to the non-uniform and non-homogeneous distribution of NPs in the GIC powder.

### 3.2. Flexural Modulus

In the current study, the highest flexural modulus was found in GIC 1 compared to GIC 2. It is interesting to note that a higher modulus was observed when nano-ZrO_2_ particles were added to GIC 1 because larger GIC glass particles were occupied by nano-ZrO_2_ particles and acted as additional bonding sites for the polyacrylic polymer, thus reinforcing the GIC matrix [31]. The strength of GICs at an early stage of setting can be affected by the nature, concentration, and molecular weight of the polycarboxylic acid; the chemical composition and microstructure of the glass; and the powder-to-liquid ratio [32]. In the current study, the flexural modulus of GIC 2 significantly declined after the incorporation of nano-ZrO_2_ particles, which might be because some larger-sized NPs leached out from the matrix, and thus their capability to bear load was decreased [33]. However, it is uncertain whether this decline was due to an unknown instrument error or an error in sample preparation or storage. It might also be due to the batch of GICs used to assess the flexural modulus in this study. The flexural modulus was not evaluated in the past with nano-ZrO_2_ particles, and therefore, there is a dearth of literature that requires more future exploration.

### 3.3. Vickers Microhardness

After 24 h of immersion in distilled water, the mean VHN of experimental groups was significantly higher than that of the control group (0%) of both the GICs. It is noteworthy that the VHN for GIC 1 is lowest (46.00VHN) at 7% and highest (55.24VHN) at 5% nano-ZrO_2_ particles. Within the matrix, the homogenous dispersion of nano-ZrO_2_ particles might be a reason for the increase in the hardness of the experimental groups. Moreover, an increase in the interaction between the NPs and the acid also leads to an increase in the hardness of the material [34]. On the contrary, in the current study, as the weight percentages of the NPs increase (7 wt% in GIC 1 and 5% weight in GIC 2), it leads to a reduction in the surface hardness of the GICs. This could be due to the agglomeration of the NPs, which were stuck to one another, leading to a decrease in surface hardness and roughness [35]. Similar results were also found when 3% (*w*/*w*) TiO_2_ NPs were added in GIC. Due to the addition of NPs, fewer glass particles were at the GIC surface, resulting in a greater amount of acid reacting with NPs. The hardness, however, decreases at higher concentrations (5% and 7% *w*/*w*) of TiO_2_ NPs due to the weakening of the bulk material [35]. The lower hardness values at 3% and 5% of nano-ZrO_2_ particles could be attributable to the formation of weak spots in the cement matrix due to voids generated by the aggregates [36]. Moreover, agglomerate accumulation on the surface, ineffective reaction between cement liquid and NPs, and the formation of voids can all decrease the hardness of the cement [36]. In this respect, useful information can be best provided by microscopic studies at the indentation sites.

### 3.4. Water Sorption and Solubility

Water sorption and solubility were determined using the International Organization for Standardization’s (ISO 9917-1:2007) [37]. In the present study, the water sorption and solubility of GIC 1 are significantly lower than the GIC 2 group. Both water sorption and solubility of GIC with nano-ZrO_2_ particles were significantly reduced at all the concentrations compared to unmodified GIC, except for solubility in GIC 1, which increased at all the concentrations. This reduction could be due to fewer porosities, which, if present, would seem shallower than those found in the unmodified cement [38]. The increase in solubility for GIC 2 at a 7% nano-ZrO_2_ concentration might be because the bond between the tooth and the restorative interface will degrade when water is incorporated at the early stage of setting. This phenomenon occurs due to two effects: degradation and lamination. Moreover, the presence of more hygroscopic fillers blended with different sizes of ultrafine, highly reactive glass particles at a 7% concentration could also be the reason for higher values of solubility. Similar values were noticed in the study conducted by Lima et al. [39]. The manual mixing of the cement, composition of the cement, and control of poor porosities are also factors supporting higher solubility. Mustafa et al. [40] presented similar results when they evaluated a resin-modified composite in comparison with a high-viscosity conventional GIC material. This difference can be explained by the more stable polymeric structure of the resin-modified composite [40]. However, there is a lack of literature regarding the water sorption and solubility of GICs. Therefore, to investigate these characteristics of restorative materials further, long-term studies are needed. Interestingly, due to different sample dimensions that affect the diffusion of water into the cement matrix, the water sorption and solubility values obtained in different studies were greatly varied. Matrix stability is decreased by smaller specimen sizes. In the current study, 10 mm × 1 mm dimensions were used for sample preparation, which is similar to the study conducted by Sunbul HA et al. [41]. However, 15 mm × 2 mm dimensions were formulated by Alrahlah et al. [42]. Regarding the water solubility of GIC 1, a statistically insignificant increase in the values was found between the control group and the modified groups with nano-ZrO_2_ particles. This increase in solubility may be caused by clumping of the NPs, resulting in a decrease in filler–matrix interaction and leading to a decrease in the intrametric homogeneity of the specimens [43]. However, statistically significant reductions were found in the solubility of GIC 2 among the modified and unmodified groups. Such differences could mean that water molecules that bind to the GIC structure after an acid–base reaction were unequal in all groups. This results in the trapping of the water molecules in the matrix, filler, or matrix–filler interface rather than being tightly bound to the structure. After drying in the desiccator, this loosely bonded water was vaporized from the samples [44]. The present study displayed negative solubility values with a large standard deviation. Many factors contribute to these negative solubility values. However, the potential reason for the negative solubility was attributed to incomplete dehydration of these materials. The negative values do not indicate that no solubility occurred, but may hint at their solubility. Previously, Toledano et al. [44] in 2006, Keyf et al. [45] in 2007, and Sinthawornkul et al. [46] in 2017 also reported negative values. The conventional GIC takes up water as the acid–base reaction progresses, and this water becomes an integral part of its structure. Therefore, the longer the rate of the reaction, the greater the water uptake into the cement structure [45]. This gain in water is retained in the cement matrix and weighted as the “final mass”. This would lead to negative solubility values and a large standard deviation of the samples. This gained water participates in a continued acid–base reaction and is not lost by the desiccation, resulting in the negative solubility values and large standard deviation of the control group in GIC 1 [45]. The incorporation of nano-ZrO_2_ particles improved both the water sorption and solubility due to many factors. In conventional GICs, over time, matrix hydrolysis results in the deterioration of cement [47]. Solubility can be reduced by adding these nanofillers, as they are water-insoluble. This aligns with the work of Dehis et al. [48], who added TiO_2_NPs into heat-cured and microwave-cured acrylic resin denture bases and compared them with conventional GICs. They found a significant reduction in water sorption and solubility. The inconsistent findings in the current study and previously conducted studies were due to different concentrations of the NPs, their size, shape, and nature, as well as methodological differences like different sizes of the samples, storage time, and solution; formulation of the water sorption; and solubility.

The nanoparticles utilized in the current study met the specific requirements and adhered to the standards as they were synthesized in our own laboratory rather than purchased commercially. This study evaluated two commercially available GICs at three different concentrations of nano-ZrO_2_ particles following a storage period of 24 h. However, the base materials, GICs, can be prepared in the laboratory by using raw materials and can be explored in future studies, which might produce stronger and more cost-effective materials than commercially available GICs. A more systematic and controlled approach is mandatory to optimize GIC reinforcement, as it will negate the compositional difference of commercially available GICs. Moreover, the long-term suitability of clinical applications of these materials can be predicted by aging GICs over an extended period, and a 24 h period is not sufficient. Nevertheless, to establish clinical correlation, further studies should be conducted by incorporating broader concentrations of nano-ZrO_2_ particles. In this work, researchers used hand-mixed GICs in which the probability of the inclusion of porosities due to air entrapment is increased, which results in an increase in the likelihood of inaccurate data. Therefore, future studies should be conducted using mechanically mixed GICs.

## 4. Materials and Methods

### 4.1. Materials

Two commercially available GICs were purchased from manufacturers for the sole purpose of this study and were used without further purification or modification. The details of each GIC are given in Table 3.

### 4.2. Methods

#### 4.2.1. Nano-ZrO_2_ Particles Preparation

The nano-ZrO_2_ particles were prepared through co-precipitation method. The methodology outlined by Foo YT et al. was followed [49].

#### 4.2.2. Characterization of Nano-ZrO_2_ Particles

Elemental morphology and average particle size of the nanocrystalline powder were evaluated via scanning electron microscope (JSM IT100, JEOL Akishima, Tokyo, JAPAN) at accelerating voltage of 10–20 kV at 10,000 magnification. The crystal phase of the specimens was determined via X-ray diffraction (XRD) technique using a Shimadzu-6000 diffractometer Shimadzu Corporation Kyoto Japan utilizing Cu K*α* (0.154 nm) radiation operating at 40 kV and 40 mA, recorded over a 2θ range of 10° to 80°.

#### 4.2.3. Incorporation of Nano-ZrO_2_ Particles into Glass Ionomer Cements

In an Eppendorf tube, nano-ZrO_2_ particles in powdered form were mixed with powder components of two different conventional GICs and then thoroughly mixed using a vortex.

#### 4.2.4. Specimens’ Preparation for Physio-Mechanical Properties

**Figure 6:**
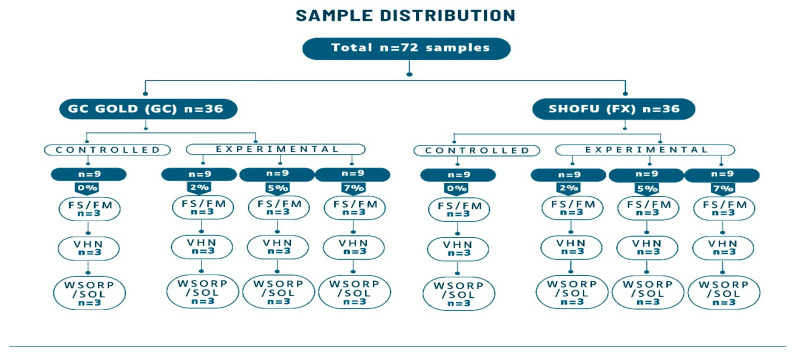
Sample distribution for GIC 1 and GIC 2. FS: flexural strength, FM: flexural modulus, VHN: Vickers hardness, WSORP/SOL: water sorption and solubility.

Seventy-two (n = 72) specimens of different sizes and shapes for different types of physio-mechanical evaluation were prepared using stainless steel molds. To determine flexural strength and flexural modulus, twenty-four (n = 24) bar-shaped specimens were used (2 × 2 × 25 mm); for Vickers microhardness, twenty-four (n = 24) disc-shaped specimens (4 × 6 mm) were used; and for water sorption and solubility, twenty-four (n = 24) disc-shaped samples (15 × 1 mm) were prepared (Figure 6). Samples were prepared at room temperature in 70% relative humidity. Powder and liquid were mixed according to the manufacturer’s recommendation and then placed in their respective stainless-steel molds. Before being removed from the mold, the specimens were left to dry at room temperature for 20 min. Prior to testing, they were coated with a protective varnish (G-C Dental Industrial Corp., Tokyo, Japan) and stored at 37 °C for 24 h in distilled water.

#### 4.2.5. Flexural Strength Evaluation (FS)

Using a universal testing machine (Testometric M500-25CT Testometric Ltd. Rochdale, United Kingdom) three point bending test was performed (ISO 4049:2009) [50] with a crosshead speed of 0.500 mm/min and preload of 0.1 N. FS (σ) was calculated using Equation (2) [51].

(2) $$\sigma =\frac{3FI}{2bh^{2}}$$

where F—the maximum load (N), l—the distance between the supports (20 mm), b—specimen’s width (2.00 mm), and h—the specimen’s height (2.00 mm)

#### 4.2.6. Flexural Modulus Evaluation (FM)

Load/displacement values produced by flexural strength testing were used to determine the flexural modulus in accordance with Equation (3) [52].

(3) $$E=\frac{p_{1}l^{3}}{4bh^{3}d}$$

where E—flexural modulus; p_1_—load in the elastic region of the stress–strain plot; l—distance between supports; h and b—the specimen’s height and width; d—deflection at p_1_.

#### 4.2.7. Vickers Hardness Evaluation (VHN)

Vickers micro-hardness tester (INDENTEC ZHV1-M) Indentec Hardness Testing, Brierley Hill, United Kingdom was used to measure the hardness. To make indentations on GIC surfaces, a 200 g load was applied for 15 s. Mean value was calculated by making three indentations with a 100 µm distance on the center of each sample.

#### 4.2.8. Water Sorption and Solubility Evaluation

After 24 h of distilled water immersion, the samples were transferred to a desiccator for 22 h and maintained at 37 °C. After this, they were kept at room temperature for an additional 2 h. Baselines dry mass (m_1_) (mg) was weighed at the end of 24 h period. The samples were then stored in 25 mL of distilled water at 37 °C for 24 h. After 24 h, the samples were removed from the distilled water. A paper towel was used to remove excess water, and the specimens were air-dried for 15 s at 23 °C before being re-weighed (m_2_). The specimens were transferred back to a desiccator, which was placed in an incubator for 22 h at 37 °C and then 2 h at 23 °C. This cycle was repeated until a constant mass was achieved (m_3_) [52,53]. The means were calculated as per Equations (4) and (5) [50,53].

(4) $$\mathrm{Water}\;\mathrm{sorption}=\frac{m_{1}-m_{2}}{V}$$

(5) $$\mathrm{Water}\;\mathrm{solubility}=\frac{m_{1}-m_{3}}{V}$$

Water sorption and solubility calculations were performed in micrograms per cubic millimeter (µg/mm^3^).

#### 4.2.9. Statistical Analysis

Minitab statistical software version 19 (Minitab 203 Ltd., Coventry, UK) was used to analyze the data. To evaluate the effect of material type (GIC 1 and GIC 2) and ZrO_2_ (0, 2, 5, and 7% *w*/*w*) on the flexural strength and modulus, micro-hardness, water sorption, and solubility, two-way analysis of variance (ANOVA) was used. To determine the differences between the means of surface hardness, flexural strength, modulus, and water sorption and solubility for the different concentrations of nano ZrO_2_ particles, one-way analysis of variance (ANOVA) and post hoc Tukey’s test were conducted for each GIC. For the comparison of all the groups, α = 0.05 was set as significance level, whereas to estimate the precision of the observed effects for the means, 95% confidence intervals were used. To highlight the effect of material type and ZrO_2_ concentrations, main effect graphs were made.

## 5. Conclusions

In this work, we developed nano-ZrO_2_ particles and incorporated them into conventional GICs. This addition enhanced the flexural strength and flexural modulus of GICs, most likely due to the reinforcement of the nano-ZrO_2_ particles by improving force transfer at the filler–matrix interface. The Vickers hardness was greatly enhanced at all concentrations for both the GICs, but at 7 wt% nano-ZrO_2_ particles, hardness was decreased due to the nanoparticles’ agglomeration. There was an increase in the water solubility of both the GICs at a 7% concentration after 24 h of water immersion as, at higher filler loading, there is a chance of incomplete matrix formation, which led to the microstructural disruption. Reduced porosity and improved matrix densification result in a significant reduction in water sorption.

## Figures and Tables

**Table 1:** Mean (standard deviation SD) of flexural strength, flexural modulus, and surface hardness of two GICs after the addition of nano-ZrO_2_ particles at varying concentrations.

Nano-ZrO_2_ Particles Present in GIC	0 wt%	2 wt%	5 wt%	7 wt%
**Mean (SD) Flexural Strength (MPa)**				
**GIC 1**	6.00 (0.4) ^c^	8.00 (0.21) **^bc^**	10.38 (1.2) **^b^**	14.00 (1.8) **^a^**
**GIC 2**	9.3 (1.3) ^a^	5.00 (0.2) **^c^**	7.00 (0.6) **^bc^**	8.2 (0.9) **^ab^**
**Mean (SD) Flexural Modulus (GPa)**				
**GIC 1**	8.41 (3.2) ^a^	8.00 (2.0) ^a^	9.41 (5.0) ^a^	12.00 (5.1) ^a^
**GIC 2**	9.0 (4.0) ^a^	7.0 (3) ^a^	3.0 (2.0) ^a^	10.00 (2.0) ^a^
**Mean (SD) Surface Hardness (VHN)**				
**GIC 1**	17.00 (0.3) ^c^	53.0 (0.3) ^a^	55.24 (1.6) ^a^	46.00 (0.2) ^b^
**GIC 2**	32.00 (5.36) ^c^	62.41 (2.39) ^a^	35.00 (1.3) **^c^**	53.00 (0.2) ^b^

Superscripts with dissimilar letters across rows for each GIC indicate statistically significant differences (*p* < 0.05).

**Table 2:** Mean (standard deviation SD) of water sorption and solubility of two GICs after the addition of nano-ZrO_2_ particles at varying concentrations.

Nano-ZrO_2_ Particles Present in GIC	0 wt%	2 wt%	5 wt%	7 wt%
**Mean (SD) Water Sorption (μg/mm^3^)**				
**GIC 1**	179.4 (56.7) ^a^	90.56 (11.88) ^ab^	122.5 (53.3) ^ab^	61.05 (2.60) ^b^
**GIC 2**	318 (178) ^a^	119.9 (30.3) ^a^	157.4 (37.4) ^a^	130 (17.8) ^a^
**Mean (SD) Water solubility (μg/mm^3^)**				
**GIC 1**	−2.52 (14.47) ^a^	15.2 (22.8) ^a^	22.22 (6.38) ^a^	5.22 (5.34) ^a^
**GIC 2**	27.3 (24.1) ^a^	15.91 (9.16) ^a^	5.66 (5.42) ^a^	35.1 (26.0) ^a^

Superscripts with dissimilar letters across rows for each GIC indicate statistically significant differences (*p* < 0.05).

**Table 3:** Composition of glass ionomer cements used in the study.

Glass Ionomer Cement	Powder	Liquid	PD/LIQ Ratio	Setting Time	Manufacturer
GIC 1	Flouroaluminosilicate glass and pigments	Polyacrylic acid, Polybasic Carboxylic acid	2.7 g:1 g	2 min 20 s	GIC CO Tokoyo Japan GC GOLD LABEL 2
GIC 2	Flouroaluminosilicate glass and pigments	Acrylic acid Tricarboxylic Acid Copolymer solution and tartaric acid	2.7 g:1 g	2 min 30 s	SHOFU INC Kyoto Japan FX Ultra shofu

## Data Availability

The original contributions presented in this study are included in the article; further inquiries can be directed to the corresponding author.

## Abbreviations

The following abbreviations are used in this manuscript:

FS	Flexural strength.
FM	Flexural modulus.
VHN	Vickers microhardness.
NPs	Nanoparticles.
